# Effects of variable nitrogen fertilization rates and dried distillers grains plus solubles supplementation on forage use, animal performance, and economic outcomes of steer calves grazing winter wheat pastures

**DOI:** 10.1093/tas/txaf116

**Published:** 2025-08-28

**Authors:** Cody E Gruber, Miriam A Snider, Michelle L Johnson, Tom Hess, Elizabeth B Kegley, J Daniel Rivera, James L Mitchell, M Shane Gadberry

**Affiliations:** Livestock and Forestry Research Station, University of Arkansas, Division of Agriculture, Batesville, AR 72501, USA; Department of Animal Science, University of Arkansas System Division of Agriculture, Fayetteville, AR 72701, USA; Livestock and Forestry Research Station, University of Arkansas, Division of Agriculture, Batesville, AR 72501, USA; Southwest Research and Extension Center, University of Arkansas, Division of Agriculture, Hope, AR 71801, USA; Department of Animal Science, University of Arkansas System Division of Agriculture, Fayetteville, AR 72701, USA; Livestock and Forestry Research Station, University of Arkansas, Division of Agriculture, Batesville, AR 72501, USA; Department of Animal Science, University of Arkansas System Division of Agriculture, Fayetteville, AR 72701, USA; Southwest Research and Extension Center, University of Arkansas, Division of Agriculture, Hope, AR 71801, USA; Department of Agricultural Economics and Agribusiness, University of Arkansas, Division of Agriculture, Fayetteville, AR 72701, USA; Livestock and Forestry Research Station, University of Arkansas, Division of Agriculture, Batesville, AR 72501, USA

**Keywords:** beef cattle, byproduct supplementation, economic returns, energy, nitrogen fertilization rates

## Abstract

With the continued increase of fertilizer prices, stocker cattle producers may utilize alternative management strategies to mitigate costs and extend the grazing season to achieve better utilization of a wheat (*Triticum aestivum* L.) crop. One potential strategy is to reduce pasture nitrogen (**N**) fertilization rates combined with animal dietary supplementation. In this 3-year (**yr**) study, wheat pastures (1.62-hectare (**ha**)) were stocked at fixed rates of 2.47 and 4.94 steer/ha in the fall and spring respectively. Nitrogen was applied as urea to experimental pastures at three different rates: 1) 100.9 (**100N**), 2) 134.5 (**135N**), and 3) 168.1 kg N/ha (**168N**). These pastures were compared to pastures with a N application rate of 100.9 kg N/ha stocked with steer calves supplemented dried distillers grains plus solubles (**DDGS**) at 1.0% body weight (**BW**; **100S**). In the spring, 100S pastures produced greater (*P* ≤ 0.05) mean and final forage mass (**FM**), as well as the greatest slope change in FM. Fall average daily gain (**ADG**) was decreased (*P* ≤ 0.05) in 168N pastures, while 100S produced greater (*P* ≤ 0.001) spring grazing days (**d**), weight gain/ha, and final BW. Both 100S and 168N produced a greater (*P ≤* 0.05) number of combined grazing d, with 100S yielding an additional 13 d. Additionally, steers assigned to the 100S treatment produced greater (*P* ≤ 0.001) combined weight gain/ha. Blood urea nitrogen (**BUN**) concentrations on D28 were greater (*P* ≤ 0.001) for steers on 100S pastures and were slightly in excess of the range for maximized growth. Total spring income/ha was greater (*P* ≤ 0.05) for 100S and 168N, with 100S making an extra $82.38/ha compared to 100N. Calf management and feed costs were greater (*P ≤* 0.001) for 100S and resulted in an increase of costs $514.60/ha above 100N. Net returns were greater (*P* ≤ 0.05) for 100N and 135N, with 100N netting the greatest returns, which averaged $361.00/ha more than the net return for 100S due to feed costs. Due to a substitution effect, an additional 525 kg dry matter (**DM**)/ha of FM was available, indicating that pastures could be stocked with an additional steer for every 2.81 ha increase in 100S pasture size, potentially providing additional economic benefits. Results from this study indicate that while DDGS supplementation provided benefits to steer and pasture performance in the spring, it was not beneficial to overall economics within the scope of the research scale production system.

## Teaser

This research demonstrates that supplementation of steer calves with dried distillers grains plus solubles on wheat pastures with lower rates of nitrogen fertilization can improve pasture and animal performance during the spring grazing season. Further research is warranted as data indicate that with greater acreage, more steers might potentially improve economic returns from supplementation.

## Graphical Abstract

**Graphical Abstract:**
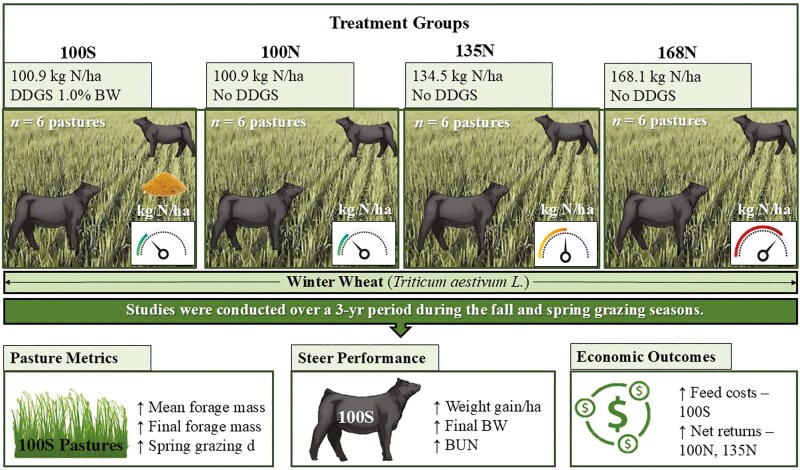

## INTRODUCTION

In recent years (**yr**), multiple inputs utilized by stocker cattle producers have substantially increased in price, with fertilizer and feedstuffs being two of the most pronounced costs. Nitrogen (**N**) fertilizer is often a necessary input to produce desired forage growth for hay production, stockpiling, or as a method to increase forage yields to increase stocking rates (Watson et al., 2012). While the demand for N fertilizers has increased, they can be costly to producers, potentially reducing net returns of fertilized pasture systems (Watson et al., 2012). Additionally, the amount of N fertilizer applied to cool-season grasses is often more than plant uptake, with a variable recovery rate between 17.0% to 50.0% (Mosier et al., 2001; Greenquist et al., 2009) contributing to potential financial losses. Although input prices have generally stabilized, large cost increases can leave stocker cattle producers with few options. Depending on the management system and production goals, producers may choose to pay the entire cost associated with fertilizing at the full required rate. If this option proves to be cost prohibitive, producers may fertilize pastures at a reduced rate and rely on subsequent forage stand responses or use conserved forages if hay prices are low enough to justify extended hay feeding. A third option of interest would be to reduce N fertilization rates while concurrently providing energetic supplements to livestock to moderate forage consumption.

During the fall and spring grazing seasons, small grain pastures have been used extensively to improve net farm income (Beck et al., 2005). In the southern United States (**U.S.**), producers will often use wheat (*Triticum aestivum* L.) for grain production and as a forage crop for cattle (Thomason et al., 2000). In small grain crops, such as wheat, N-based fertilizers play important roles in replenishing plant nutrients, such as grain protein content, as well as improving forage yield quantity and nutritive value (Tabak et al., 2020). Wheat serves as an excellent forage source for growing beef steers, as the crude protein (**CP**) and total digestible nutrient (**TDN**) content both exceed animal nutritional requirements. As a result, limitations in animal performance generally stem from limited forage availability (Beck and Reuter, 2022). For animal performance to be maximized when grazing wheat, a forage allowance of 1.59 kg of forage DM/kg of steer body weight (**BW**) has been shown to maximize average daily gains (**ADG**) at 1.22 kg/d (**d**; Beck and Reuter, 2022). Additionally, forage mass (**FM**) of wheat pastures has been shown to become limiting for growing calves when forage availability falls below 1,000 kg DM/hectare (**ha**; Redmon et al., 1995; Morgan et al., 2012).

As protein concentrations in wheat are greater during the fall and early spring, the TDN:CP ratio is often imbalanced with available energy being inadequate (Beck and Reuter, 2022). Supplementation with energy-based feeds, such as cereal grains or digestible byproducts, are often used to correct this energetic imbalance for cattle grazing wheat pastures by providing rumen available energy. Dried distillers grains plus solubles (**DDGS**) is a byproduct feedstuff that is widely available and has remained a popular option due to its high TDN and CP content (Klopfenstein et al., 2008; Gunter et al., 2021). This is primarily due to the removal of starch during the ethanol production process, increasing the concentration of other nutrients (Spiehs et al., 2002; Adams et al., 2022). Although wheat regularly produces CP values greater than 25.0% in early vegetative growth, DDGS has the potential to balance out protein and energy supplies as plant nutritive value diminishes with maturity. Additionally, supplementation of stockers grazing annual ryegrass (*Lolium multiflorum* Lam.) with 56.0 kg N/ha with DDGS has been shown to yield greater ADG than steers grazing annual ryegrass fertilized with 112.0 kg N/ha, indicating this N delivery method may be more effective than increased fertilization rates (Gunter et al., 2021). Furthermore, supplementation with DDGS may make stocker economic performance more predictable by increasing BW gains to improve overall economic performance (Horn, 2006).

Supplementing grazing cattle with DDGS may act as a N fertilizer as excess N is excreted (Greenquist et al., 2011). Greenquist et al. (2009) showed that supplementing yearling cattle with 2.3 kg DDGS/ hd, daily at amounts of 0.58% BW/d resulted in a N fertilization rate of 35.0 kg/ha—40.0 kg/ha. Based on this research, supplementation of cattle grazing winter annual forages with DDGS in place of utilizing a full fertilization rate presents a potential opportunity to capitalize on price differences between N fertilizer and DDGS at that point in time. We hypothesized that DDGS supplementation would improve both animal and pasture performance, potentially improving economic efficiency. Therefore, the objective of this study was to evaluate steer, pasture, and economic performance of stocker cattle grazing wheat fertilized with three levels of N fertilizer and an additional treatment where the lowest N fertilizer rate included steers being supplemented DDGS at 1.0% BW.

## MATERIALS AND METHODS

This project and all procedures were approved by the University of Arkansas Institutional Animal Care and Use Committee (#21139). This study was conducted at the University of Arkansas Division of Agriculture Livestock and Forestry Station (**LFST**) stocker unit (35.82 N, -91.79 W) near Batesville, AR from Fall 2021—Spring 2024.

### Weather Conditions

As previously described by Gadberry et al. (2024), the LFST operates as an official reporting station for the National Weather Service (Station ID: USC00030458). National Weather Service precipitation and temperature data for Fall 2021—Spring 2024 and historical data (1941 – 2021) were available through the National Oceanic and Atmospheric Administration (**NOAA**) online climate data portal (NOAA, 2024). Average ambient temperatures (°C) and cumulative monthly precipitation (mm) were collected from September to April for each yr during the three-yr study. These values were compared to 80-yr temperature and cumulative precipitation averages from the LFST.

### Site Description and Pasture Establishment

Soils were predominately a Peridge silt loam soil type and with a 3.0% to 8.0% slope (USDA-NRCS, 2024) and had previously been utilized for wheat grazing systems research. A total of 24 pastures 1.62-ha in size were used in Yr 1 (Fall 2021—Spring 2022) and 2 (Fall 2022—Spring 2023). Pasture size was held constant for the entirety of the study. Four pastures were removed during Yr 3 (Fall 2023—Spring 2024) due to poor stand performance and insufficient available forage (*n *= 20 pastures). All pastures were established using a no-till (**NT**) method. Fields were sprayed with glyphosate in late August at a rate of 4.67 L/ha using an 1,892.71-L Wylie Pull-Type Sprayer (Wylie Sprayers, Lubbock, TX) to induce a chemical fallow prior to planting. Pastures were planted with 112.09 kg/ha of a ‘Hilliard’ cultivar of soft red winter wheat (Petrus Seed & Grain Company, Inc., Hazen, AR) during the first week (**wk**) of September using a John Deere 750 NT Seed Drill (John Deere, Moline, IL) or a Haybuster 107C drill (Haybuster, Jamestown, ND). Fertilizer was applied yearly with a split application with the first application occurring following fall planting and the second occurring during the spring when daytime average temperatures trended above 10 °C. Nitrogen fertilizer was applied as urea at the rate corresponding with each field’s respective treatment by broadcasting using variable rate application.

### Steer Receiving Period

Over a three-yr period, 816 preconditioned *Bos taurus* crossbred beef steers (267.41 ± 40.25 kg) were utilized in this study, with the majority of steers acquired from a cooperating producer. Cattle arrived three to four wk before each trial period began (**Table 1**). Ten additional steers were also acquired from the same source to serve as replacements in the event of steer death or removal from the study for health reasons. Following arrival, steers were weighed and commingled in receiving pens. Steers were provided either pearl millet (*Pennisetum glaucum* (L.) R.Br.) baleage or grass hay depending on availability. Cattle had ad libitum access to water, a complete mineral mix containing an ionophore (same mineral provided during grazing), and DDGS supplemented at 0.5% of BW on an as-fed basis. Pour-on dewormer (Cydectin, Elanco, Greenfield, IN) and a growth implant (40.0 mg trenbolone acetate and 8.0 mg estradiol, Component TE-G, Elanco, Greenfield, IN) were administered to each steer before turnout. Following arrival and prior to turnout, all steers were stratified and blocked by weight to maintain similar total pasture BW across pastures. Steers were then randomly allocated to pastures that were previously assigned random treatments prior to the beginning of the study.

**Table 1.** Receiving, grazing initiation, and grazing termination dates of steer calves grazing wheat pasture stands of variable **nitrogen (N)** fertilization rates with or without dried distillers grains plus solubles (**DDGS**) supplementation over a three-year (**yr**) period

			Treatments^1^			
Study Yr	Season	Item	100S	100N	135N	168N
Yr 1	Fall 2021	Steers	*n *= 96			
		Receiving	Oct 20 – 21	Oct 20 – 21	Oct 20 – 21	Oct 20 – 21
		Grazing initiation	Nov 17	Nov 17	Nov 17	Nov 17
		Grazing termination	Dec 15—Feb 7	Jan 5—Feb 7	Dec 15—Jan 13	Dec 15—Feb 7
		Grazing d range	28 – 82	49 – 82	28 – 82	28 – 82
	Spring 2022	Steers	*n *= 192^2^			
		Receiving	Feb 9	Feb 9	Feb 9	Feb 9
		Grazing initiation	Mar 8	Mar 8	Mar 8	Mar 8
		Grazing termination	May 3	Apr 5—May 3	Apr 5—May 3	Apr 5—May 3
		Grazing d range	56	28 – 56	28 – 56	28 – 56
Yr 2	Fall 2022	Steers	*n *= 96			
		Receiving	Oct 12	Oct 12	Oct 12	Oct 12
		Grazing initiation	Nov 21	Nov 21	Nov 21	Nov 21
		Grazing termination	Jan 26	Jan 10—Jan 26	Jan 26	Jan 26
		Grazing d range	66	50 – 66	66	66
	Spring 2023	Steers	*n *= 192			
		Receiving	Feb 16	Feb 16	Feb 16	Feb 16
		Grazing initiation	Mar 1	Mar 1	Mar 1	Mar 1
		Grazing termination	May 10	Apr 12—May 10	Apr 12—May 10	Apr 19—May 3
		Grazing d range	70	42 – 70	42 – 70	49 – 63
Yr 3	Fall 2023	Steers	*n *= 80			
		Receiving	Oct 24	Oct 24	Oct 24	Oct 24
		Grazing initiation	Dec 6	Dec 6	Dec 6	Dec 6
		Grazing termination	Jan 11—Jan 17	Jan 11—Jan 17	Jan 11—Jan 17	Jan 17
		Grazing d range	36 – 42	36 – 42	36 – 42	42
	Spring 2024	Steers	*n *= 160^3^			
		Receiving	Feb 13	Feb 13	Feb 13	Feb 13
		Grazing initiation	Mar 4	Mar 4	Mar 4	Mar 4
		Grazing termination	Apr 29	Apr 15—Apr 29	Apr 29	Apr 29
		Grazing d range	56	42 – 56	56	56

^1^Treatments consisted of: 1) 100.9 kg N/ha + DDGS supplementation (100S; *n* = 6 pastures/season), 2) 100.9 kg N/ha (100N; *n* = 6 pastures/season), 3) 134.5 kg N/ha (135N; *n* = 6 pastures/season), and 4) 168.1 kg N/ha (168N; *n* = 6 pastures/season) in Yr 1 and Yr 2. In Yr 3, pastures were reduced to *n *= 5 pastures/treatment/season due to poor stand performance and insufficient available forage.

^2^Sixty-eight steers were retained from Fall 2021 and 47 steers utilized were station-owned.

^3^Fourteen steers were retained from Fall 2023.

### Treatments and DDGS Supplementation

As stated previously, a total of 24, 1.62 ha pastures were utilized in this study. In Yr 1 and Yr 2, pastures (*n *= 18) were divided into three N fertilizer application rates: 1) 100.9 kg N/ha (**100N**), 2) 134.5 kg N/ha (**135N**), and 3) 168.1 kg N/ha (**168N**). Steers assigned to these pastures were not provided with DDGS supplementation. Additional pastures (*n* = 6) were fertilized at the low N rate (100.9 kg N/ha) and provided DDGS daily (**100S**). In Yr 3, pastures were reduced to *n *= 5 pastures/treatment/season due to poor stand performance. Dried distillers grains plus solubles were sourced from a local feed supplier in Batesville, AR and supplemented once daily at 0900 hr to steers grazing 100S pastures at a rate of 1.0% of BW on an as-fed basis. Amounts of DDGS provided to 100S paddocks were adjusted following weigh dates based on most recent steer BW. Orts were collected in instances of feed refusal. Samples of DDGS were collected weekly and pooled throughout study period within study yr. Nutrient analysis of the pooled sample within study yr was conducted via wet chemistry analysis at a commercial laboratory (Dairy One, Ithaca, NY; **Table 2**) using the following methods: DM was determined by drying in an oven for two hr at 135 °C (NFTA method 2.2.1.1). Crude protein was analyzed following methods 990.03 (AOAC International, 2006). Acid detergent fiber (**ADF**; ANKOM technology method 14, 2020) was analyzed after digestion in ADF solution and in an acetone soak and neutral detergent fiber (**NDF**) was analyzed using ANKOM technology method 15. Ether extract (**EE**) was analyzed using ANKOM technology method 2. Macromineral (Ca, P, Mg, K, Na, S) content and trace mineral (Fe, Zn, Cu, Mn, Mo) content were analyzed using the CEM microwave digestion method. Total digestible nutrients were estimated using equations from the NRC (NRC, 2001) and non-fiber carbohydrates (**NFC**) were calculated from wet chemistry components.

**Table 2.** Nutritive value of dried distillers grains plus solubles (**DDGS**) supplemented to steers grazing wheat pastures fertilized with nitrogen (**N**) at a rate of 100.9 kg N/hectare (**ha**) over a three-year (**yr**) period

	Grazing Season			
Item	Yr 1 Fall 2021—Spring 2022	Yr 2 Fall 2022—Spring 2023	Yr 3 Fall 2023—Spring 2024	Normal Nutrient Content Ranges^1^
DM^2^, %	88.2	87.3	87.7	84.2 – 97.0
% DM				
CP^3^	31.8	33.1	33.5	26.7 – 35.8
ADF^4^	13.6	12.4	13.2	13.2 – 19.7
NDF^5^	37.2	33.6	34.6	29.6 – 38.7
NFC^6^	16.0	17.5	17.4	15.5 – 30.3
EE^7^	8.8	8.0	8.7	8.1 – 14.7
Ash^8^	6.2	7.8	5.8	4.9 – 7.7
TDN^9^	79.0	78.0	80.0	76.0 – 87.0
Minerals^10^, % DM				
Ca	0.13	0.12	0.07	0.00 – 0.28
P	1.20	1.28	1.10	0.72 – 1.08
Mg	0.35	0.36	0.35	0.25 – 0.41
K	1.31	1.49	1.29	0.81 – 1.40
Na	0.443	0.379	0.422	0.013 – 0.415
S	0.85	0.85	0.64	0.21 – 1.11

^1^Normal DDGS nutrient content ranges as defined by Dairy One (Ithaca, NY).

^2^Dry matter (**DM**); analyzed using National Forage Testing Association (**NFTA**) method 2.2.1.1.

^3^Crude protein (**CP**); analyzed using AOAC official method 990.03.

^4^Acid detergent fiber (**ADF**); analyzed using ANKOM technology, method 14.

^5^Neutral detergent fiber (**NDF**); analyzed using ANKOM technology, method 15.

^6^Non-fiber carbohydrates (**NFC**); analyzed via wet chemistry methods.

^7^Ether extract (**EE**); analyzed using ANKOM technology, method 2.

^8^Analyzed using AOAC official method 942.05.

^9^Total digestible nutrients (**TDN**) calculated using National Research Council Nutrient Requirements of Dairy Cattle, 7^th^ ed. (NRC, 2001) equations.

^10^Analyzed using CEM microwave digestion method.

### Steer Management and Animal Data Collection

Steers were individually weighed at turnout, every 28 d, and upon removal from pasture to assess initial BW, final BW, and weight gain. Cattle were turned out on pasture once FM was deemed sufficient for grazing (**Table 1**). Grazing was concluded when FM became limiting at less than 1,150 kg/ha or when excessive plant reproductive maturity resulted in insufficient plant nutritive value in spring (Gadberry et al., 2004). Steers were stocked at a fixed rate of 2.47 steer/ha and 4.94 steer/ha in the fall and spring respectively to account for increased FM accumulation due to warmer spring temperatures and wheat entering the reproductive stage of growth. Forage allowance for both fall and spring grazing seasons were calculated using the following calculation:


$$\begin{array}{l} FM/ha\;\;\;\left(kg\;\;\;DM/ha\right)\;\;\;÷\;\;\;(stocking\;\;\;rate\;\;\;\times average\;\;\;BW/hd\;\;\;\left(kg\;\;\;BW/ha\right). \end{array}$$

Blood samples for blood urea nitrogen (**BUN**) were collected on D0 and D28 during the spring of Yr 3 (*n* = 104 steers). Briefly, samples were collected via jugular venipuncture with an 18G needle into 10 mL red-top vacutainer (BD, Franklin Lakes, NJ). Blood was centrifuged at 1700 x *g* at 4 °C for 20 min. Serum was collected, stored at -20 °C until analysis, and analyzed for BUN concentrations via a commercially available colorimetric kit (TECO Diagnostics, Anaheim CA). Assays were performed in a flat-bottom 96-well plate with a 6x dilution used to bring sample concentrations within standard curve range. Samples were analyzed in duplicate on a microplate reader (Biotek EPOCH, Biotek Instruments Inc., Winooski, VT) at a ʎ of 340 nm. The intra- and inter-assay coefficients of variation were 1.6% and 3.0%, respectively.

All steers were provided free choice access to a mineral supplement throughout the grazing period. In Yr 1 steers were provided Growth-Booster Free Choice Bovatec Medicated Mineral (Ragland Mills, Inc., Neosho, MO; Alpharma, Bridgewater, NJ) with Bovatec provided at 200 mg steer/d. In Yr 2, Fortigraze Wheat/Rye Medicated Mineral (Livestock Nutrition Center, Chickasha, OK) was provided. In Yr 3, MasterGain Wheat Pasture Mineral (ADM Animal Nutrition, Quincy, IL) was offered with monensin provided at 150 mg steer/d. Minerals differed by yr due to availability from feed suppliers. Mineral was checked every 2 wk and refilled based on disappearance.

### Forage Sampling and Measurements

Pasture sampling corresponded with initiation of grazing, at the 28-d steer weigh-date intervals (± 3 d-intervals in the event of inclement weather), and the date of removal of steers from pastures. Pasture sampling consisted of FM measurements using a calibrated rising plate meter, FM calibration samples, and forage quality samples obtained every sample date. Additionally, plant stand counts and plant height measurements were collected on the turnout date of every fall period. Forage quality samples were collected by hand at 3 to 4 random spots in each pasture to obtain a representative quality sample of each individual pasture. Samples were subsequently placed in paper sacks, dried in a forced air oven at 55 °C, and stored at room temperature until near-infrared reflectance spectroscopy (**NIRS**) analysis could be conducted.

To measure FM, 20 readings/pasture were collected using a calibrated rising-plate meter (Michell and Large, 1983; Beck et al., 2022). Using a rising plate meter, a calibration curve for FM estimation was created. Briefly, calibration samples were collected from 15 pastures every sample date and were collected within a 0.25 m^2^ polyvinyl chloride square. Pasture samples were clipped to a residual height of less than 5-cm using battery-powered handheld clippers. Samples were stored in paper sacks and dried in a forced-air oven at 55 °C for 48 hr with the resulting DM weighed. Plate meter calibration curves were created for both fall and spring seasons for each yr of the study. Regression models included both intercept and quadratic forms of plate meter height; however, non-significant coefficients were removed from the prediction equations. The minimal coefficient of determination among the fitted models was 0.88.

Plant stand assessments were completed through visual appraisal before the start of grazing in fall of Yr 1. Before fall turnout in Yr 2 and Yr 3, plant stand counts were measured as an assessment of percent coverage. ArcGIS Pro (Esri, Redlands, CA) was used to randomly select plant canopy coverage locations where a 5 × 5 grid would be placed to score presence or absence of wheat within each grid.

### Forage Nutrient Analysis

Prior to nutritive analysis, pasture forage samples were ground to a 2-mm grind size using a Thomas-Wiley Model 4 Laboratory Mill (Thomas Scientific, Swedesboro, NJ) fitted with a 2-mm screen. Samples were stored at room temperature prior to nutrient analysis. Nutrient analysis was conducted at the forage lab at the University of Arkansas Southwest Research and Extension Center (Hope, AR) using NIRS (FOSS, Hilleroed, Denmark). Forage nutritive content values, including CP, NDF, and ADF reported on a DM-basis based on the methods of Beck et al. (2019).

### Economic Assessment

The value of steers at turnout and removal from pasture was based on the corresponding USDA weekly market reports for Arkansas. The Mississippi State Budget Generator (Mississippi State University Department of Agricultural Economics, 2024) was used to generate direct and fixed expenses related to establishment of wheat pasture. Actual expenses were used each yr for fertilizer, chemical, feed, and mineral. Veterinary medical expenses were based on recorded drug usage and corresponding product cost. Economic returns did not include all costs relevant to producers, with variable costs such as interest on operating loans, pasture rental rates, and fixed investment costs being omitted. As a result, relative economic returns are compared across treatments, as opposed to absolute economic returns.

### Statistical Analysis

All treatment response data were analyzed using the mixed model procedure of JMP 17 (SAS Inst. Inc., Cary, NC). Year was considered a random effect, and pastures were the experimental units. Data was aggregated to the pasture level prior to statistical analysis. All responses were analyzed by season except the economic analysis. Analysis by season was conducted to account for differing stocking rates and growing conditions between seasons, where fall produced more vegetative growth under drier conditions while spring produced more reproductive forage growth under wetter conditions. A combined season analysis was performed for total grazing d, total gain/ha, and economic responses. A Dunnett’s test was performed with the DDGS treatment set as the control and compared to each non-supplemented N rate treatment since our initial objective was to determine if supplementation at the lowest N fertilization rate would yield animal and pasture performance results similar to either of the increased N fertilization rates. An additional N fertilization rate contrast for linear or quadratic responses was not included in the current analysis as it is a component of an additional dataset studying the interaction of N fertilization rate and establishment method.

Two calves from 135N treatment pastures died in Fall 2021 (Yr 1), while one calf from the 100S treatment was removed due to injury in Fall 2022 (Yr 2). Because of the low incident rate and inconsistent association with treatment, losses were considered independent of treatments and should not reflect on the estimated treatment outcomes. Therefore, these calves were replaced with extra “grazers” of similar weight (*n *= 3) to maintain each pasture’s original stocking rates. However, substitute “grazer” performance was excluded from the analysis since they were not on the assigned treatment for the entire duration of the grazing season. For pastures that received a substitute “grazer,” only the three original steers from affected pastures were used to summarize per steer response data. Pasture response totals, such as gain/ha, were calculated using the summarized per steer response multiplied by the stocking rate.

Steer responses were aggregated to the pasture level prior to analysis and dependent variables analyzed included initial BW, final BW, ADG, and BUN concentrations. As mentioned previously, BUN was only measured during a single season of a single year; therefore, the year random effect was excluded from the BUN model. Responses measured at the pasture level were grazing d, and weight gain/ha. Forage responses consisted of initial FM, ending FM, average FM, and the slope change (coefficient) in FM. The slope coefficient was estimated for each pasture within season and study year using a linear regression model with the estimated kg DM/pasture as the dependent variable and d since initiation of grazing as the independent variable. The slope coefficient from each pasture within year and season was then analyzed for treatment effect and Dunnett comparison in a similar manner as other response variables analyzed. Economic responses were analyzed on a per ha basis, with economic dependent variables consisting of income (total fall income and total spring income), expenses (initial fall value, initial spring value, establishment cost, fertilizer, chemical, veterinary care/medicine), a feed-related cost assessment (feed cost and feeding labor cost), and overall net return.

## RESULTS AND DISCUSSION

### Weather Conditions and Pasture Performance

With fertilizer costs remaining high, determining the fertilization rate that elicits a maximized forage growth response without wasting input is important to maintaining cost-effectiveness within a stocker cattle operation. In general, kg of available FM can be expected to increase linearly in cool-season forage systems as the amount of applied N is increased and can also be expected to decrease linearly as applied N is decreased (Blaser, 1964; Reid, 1966; Gunter et al., 2019). However, additional environmental factors also play a role in wheat stand performance and may negate any expected performance increases from additional N input.

On average, cumulative monthly precipitation was generally lower than historical trends with a few exceptions. Above average total precipitation was observed in January and April of Yr 1, December through March in Yr 2, and October and January in Yr 3 (**Table 3**). Fall grazing seasons for all study yr were generally drier on average as indicated by the prolonged below average precipitation from September to November. Spring grazing began with more precipitation than the historical average as shown by increased amounts of precipitation in January before generally becoming drier with the exception of Yr 2. Decreased precipitation leading into and throughout the fall grazing season may explain the delayed start of grazing for Fall 2023 (Yr 3) in which the onset of grazing was delayed by 15 d from the yr prior, and 19 d from Fall 2021 (Yr 1).

**Table 3.** Monthly cumulative precipitation (mm) and average daily temperatures (°C) during the fall and spring growing seasons at the Livestock and Forestry Research Station (**LFST**) near Batesville, AR as compared to 80-year (**yr**) historical averages (1941 – 2021).

	Livestock and Forestry Research Station, Batesville, AR		
Month	Yr 1^1^	Yr 2	Yr 3
	Monthly cumulative precipitation, mm		
September	71.4 (-17.2)^2^	12.2 (-76.4)	49.8 (-38.8)
October	68.5 (-22.0)	56.4 (-34.1)	125.9 (35.4)
November	60.0 (-64.0)	90.6 (-33.4)	34.9 (-89.1)
December	118.2 (-3.8)	155.0 (33.0)	80.6 (-41.4)
January	184.1 (53.1)	187.0 (56.0)	200.8 (69.8)
February	64.7 (-72.3)	150.2 (13.2)	46.5 (-90.5)
March	122.5 (-12.5)	253.0 (118.0)	80.3 (-54.7)
April	138.1 (18.1)	72.4 (-47.6)	70.9 (-49.1)
	Average daily temperature, °C		
September	22.1 (0.2)	21.2 (-0.7)	21.4 (-0.5)
October	16.8 (0.7)	14.3 (-1.8)	16.1 (0.0)
November	8.1 (-1.6)	8.1 (-1.6)	9.2 (-0.5)
December	10.0 (5.5)	3.5 (-1.1)	6.5 (2.0)
January	1.4 (-1.7)	5.8 (2.7)	-0.2 (-3.3)
February	3.6 (-1.8)	7.3 (1.9)	9.4 (4.0)
March	9.6 (-0.5)	9.0 (-1.1)	11.3 (1.2)
April	13.5 (-2.1)	14.2 (-1.4)	15.6 (0.0)

^1^Yr 1: Fall 2021—Spring 2022; Yr 2: Fall 2022—Spring 2023; Yr 3: Fall 2023—Spring 2024.

^2^Departures from historical averages are indicated in parentheses. Negative values within parentheses indicate lower amounts of precipitation and lower temperatures compared to the historical average. Positive values within parentheses indicate higher amounts of precipitation and higher temperatures compared to the historical average.

To assess pasture performance, measurements of change in FM over time (slope coefficient) as well as initial, final, and mean FM were collected in the fall and spring for the duration of the study. During the fall grazing season, a trend (*P *= 0.06) of greater final FM for 100S pastures was observed (**Table 4**). There were no differences in initial or mean FM observed between treatments during the fall grazing season. Furthermore, there were no differences for the slope coefficient between the 100S treatment and all other treatments. However, FM of pastures in all treatments began to decline as indicated by the negative slope. Differences in FM and slope were more apparent in the spring grazing season. Pastures receiving supplementation (100S) produced greater mean and final FM (*P* ≤ 0.05) compared to pastures stocked with unsupplemented steers, regardless of N fertilization rate. Additionally, 100S pastures had the greatest slope change in FM (8.5 kg DM/ha; *P *≤ 0.001), indicating that FM was accumulating rather than diminishing. While 100S produced the greatest slope change, 135N and 168N also produced positive slope change values of 0.4 kg DM/ha and 2.1 kg DM/ha respectively (*P *< 0.001). The 100N treatment pastures yielded a negative slope change value of -3.7 kg DM/ha, indicating diminishing FM over the season

**Table 4.** Average forage mass (**FM**) of wheat stands fertilized with variable rates of nitrogen (**N**)^1^ with and without steer calves supplemented with dried distillers grain plus solubles (**DDGS**)^2^

	Treatments^3^ (kg/ha)					
Item	100S^4^	100N	135N	168N	SEM	*P*-value^5^
Fall Grazing Season						
FM, kg DM/ha						
Initial	1,696.0	1,547.0	1,482.0	1,583.0	158.0	0.36
Final	1,198.0^c^	1,048.0^d^	1,185.0^d^	1,134.0^d^	82.9	0.06
Mean	1,499.0	1,301.0	1,350.0	1,378.0	96.4	0.20
Slope change^6^	-8.0	-9.3	-6.5	-7.9	2.61	0.26
Spring Grazing Season						
FM, kg DM/ha						
Initial	1,101.0	949.0	1,022.0	976.0	179.0	0.12
Final	1,489.0^a^	755.0^b^	1,046.0^b^	1,115.0^b^	171.0	<0.001
Mean	1,476.0^a^	951.0^b^	1,189.0^b^	1,177.0^b^	87.9	<0.001
Slope change^6^	8.5^a^	-3.7^b^	0.4^b^	2.1^b^	5.72	<0.001

^1^Nitrogen (**N**) fertilization treatments were applied yearly and split between 50% application at planting and the remaining 50% at spring green up.

^2^Dried distillers grains plus solubles (**DDGS**) supplemented at 1.0% of steer body weight (**BW**).

^3^Treatments consisted of: 1) 100.9 kg N/ha + DDGS supplementation (100S; *n* = 6 pastures/season), 2) 100.9 kg N/ha (100N; *n* = 6 pastures/season), 3) 134.5 kg N/ha (135N; *n* = 6 pastures/season), and 4) 168.1 kg N/ha (168N; *n* = 6 pastures/season) in Yr 1 and Yr 2. In Yr 3, pastures were reduced to *n *= 5 pastures/treatment/season due to poor stand performance and insufficient available forage.

^4^100S treatment was set as a control within Dunnett’s test.

^5^F-test value of analysis of variance.

^6^Slope change was estimated by linear regression of forage mass (**FM**) among sampling dates.

^a-b^Within row, means with different superscripts differ at *P* ≤ 0.05 of N levels compared to Dunnett’s control.

^c-d^Within row, means with different superscripts differ at *P* ≤ 0.1 of N levels compared to Dunnett’s control.

While seasonal patterns and N application play a role in FM production, a substitution effect, where supplemented steers consume less forage due to intake of supplemental feed, may have contributed to increased final (fall and spring) and mean (spring) FM in 100S pastures as compared to pastures with unsupplemented steers. Research has shown that supplementation of DDGS to grazing cattle may replace forage at 0.27 to 0.79 kg/kg DDGS supplemented (Griffin et al., 2009). The results of the current study align with those of Loy et al. (2007) and MacDonald et al. (2007) who both observed a decrease in forage intake when dried distillers grains (**DDG**) were supplemented to heifers that were either fed chopped grass hay (Loy et al, 2007) or grazing smooth bromegrass paddocks (MacDonald et al., 2007). In the current study, results are in agreeance with that of Moore et al. (1999) who indicated that voluntary forage intake was shown to decrease when supplements were provided to cattle grazing improved pasture, while supplementation to cattle grazing native grasses or straw increased intake. Supplementation of DDGS to growing cattle on wheat pasture likely reduced voluntary forage intake to a point where slope change values indicated that forage was accumulating during a period where unsupplemented pastures showed diminished FM. This indicates that recycled N potentially benefitted forage growth, a substitution of forage intake occurred, or that there was a synergistic effect.

### Forage Nutrient Composition and Forage Allowance

The initial fall CP of wheat was greater (*P* = 0.04) for 168N pastures while initial NDF and ADF were lower (*P ≤ *0.05). This indicates that 168N pastures may have initially been of a greater nutritive value than pastures receiving the lowest N application rates (**Table 5**). However, initial CP content of wheat stands in all pastures was at or above the typical CP content of wheat and other cool-season annual grasses (> 25% CP DM-basis; Beck and Reuter, 2022). Average initial NDF and ADF for all fall pastures were lower than the typical ranges described by Beck and Reuter (2022) of 40.0% to 49.0% NDF and 19.0% to 29.0% ADF. These values indicate that pasture nutritive value was improved in the beginning of the growing and grazing seasons with a high CP content and low fiber content. Nutrient composition of wheat pastures in the fall for final CP, NDF, and ADF were not affected by level of N fertilization or DDGS supplementation. However, as expected, numerically CP decreased and fiber content increased as the season progressed indicating a decline in forage nutritive value. Average NDF and ADF content of wheat at the end of the grazing season were still within commonly accepted ranges (Beck and Reuter, 2022). No differences were observed during the spring for initial or final CP, NDF, and ADF. Similar to fall forage nutritive values, average initial CP was greater than 25.0% on a DM-basis. It should be noted that comparing the 100S and 100N responses, nutrients added to the system from supplementation did not appear to influence measures of nutritive value as carry-over from fall to spring.

**Table 5.** Average nutrient composition of wheat stands fertilized with variable rates of nitrogen (**N**)^1^ with and without steer calves supplemented with dried distillers grain plus solubles (**DDGS**)^2^.

	Treatments^3^ (kg/ha)					
Item^4^ (% DM)	100S^5^	100N	135N	168N	SEM	*P*-value^6^
Fall Grazing Season						
CP^7^						
Initial	25.3^b^	24.9^b^	25.6^b^	26.7^a^	1.80	0.04
Final	22.0	20.6	21.3	21.9	2.24	0.32
NDF^8^						
Initial	33.7^a^	33.6^a^	33.5^a^	31.6^b^	2.60	0.04
Final	46.7	47.6	46.6	47.1	2.14	0.93
ADF^9^						
Initial	16.3^a^	16.8^a^	16.1^a^	15.3^b^	1.67	0.02
Final	25.7	27.1	25.9	26.4	2.30	0.79
Spring Grazing Season						
CP						
Initial	29.1	28.0	28.3	29.3	1.57	0.20
Final	14.7	16.1	14.2	15.0	1.71	0.29
NDF						
Initial	39.7	40.5	41.3	39.6	2.92	0.30
Final	59.3	57.8	56.2	56.3	2.27	0.32
ADF						
Initial	20.7	21.5	21.7	20.8	2.36	0.38
Final	35.5	35.2	33.7	33.9	1.10	0.49

^1^Nitrogen (**N**) fertilization treatments were applied yearly and split between 50% application at planting and the remaining 50% at spring green up.

^2^Dried distillers grains plus solubles (**DDGS**) supplemented at 1.0% of steer body weight (**BW**).

^3^Treatments consisted of: 1) 100.9 kg N/ha + DDGS supplementation (100S; *n* = 6 pastures/season), 2) 100.9 kg N/ha (100N; *n* = 6 pastures/season), 3) 134.5 kg N/ha (135N; *n* = 6 pastures/season), and 4) 168.1 kg N/ha (168N; *n* = 6 pastures/season) in Yr 1 and Yr 2. In Yr 3, pastures were reduced to *n *= 5 pastures/treatment/season due to poor stand performance and insufficient available forage.

^4^Samples were analyzed using near-infrared reflectance spectroscopy (**NIRS**).

^5^100S treatment was set as control within Dunnett’s test.

^6^F-test value of analysis of variance.

^7^Crude protein (**CP**).

^8^Neutral detergent fiber (**NDF**).

^9^Acid detergent fiber (**ADF**).

^a-b^Within row, means with different superscripts differ at *P* ≤ 0.05 of N levels compared to Dunnett’s control.

Fall FM (expressed on a per kg BW-basis) was not affected (*P* ≥ 0.19) by treatment at initiation of grazing (2.2 ± 0.34 kg DM/kg BW) or at the conclusion of grazing (1.3 ± 0.17). However, 100S was numerically greater than 100N by 0.14 kg FM/kg BW at the conclusion of fall grazing. Spring FM when expressed on a per kg of BW differed initially in spring (*P* = 0.02) with the 100S treatment measuring 0.11 to 0.17 ± 0.1 kg FM/kg BW greater than the 3 N rate treatments. After adjusting for initial spring FM per kg BW, FM at the conclusion of grazing was 0.32, 0.21, and 0.27 ± 0.06 kg FM/kg BW greater for 100S compared to 100N, 135N, and 168N, respectively. Spring FM averaged 0.86, 0.63, 0.72, and 0.66 ± 0.087 kg FM/kg BW for 100S, 100N, 135N, and 168N, respectively. It should be noted that the average FM in the fall was approximately 44% greater than the average FM in spring when expressed on a per kg BW basis. This may have contributed to observing more treatment responses in spring relative to fall.

### Steer Growth and Performance

In this study, the ADG of steers assigned to the 100S treatment (1.38 kg/d) did not differ from the ADG of steers assigned to the 100N (1.30 kg/d) or 135N (1.26 kg/d) treatments during the fall grazing season (*P *> 0.05; **Table 6**). However, steers assigned to the 168N treatment had a decreased ADG (1.25 kg/d; *P* ≤ 0.05) during the fall grazing season compared to the 100S treatment despite initial and mean FM, grazing d, and final forage nutrient content (*P *> 0.05) not differing from the other treatments. Higher N fertilization rates in pastures generally lead to increased ADG of grazing cattle when pastures are fertilized within a certain range of N. Research has shown that optimal N fertilization depends on animal management and climate factors. For example, Gunter et al. (2005) evaluated the effects of stocking rate and N fertilization rates on steers grazing primarily dallisgrass (*Paspalum dilatatum* Poir.) pastures. It was found that the ADG of steers increased as more N fertilizer was applied to pastures; however, ADG decreased in response to increased stocking rate. Berg and Sims (1995) examined the impacts of N fertilization rate with a variable stocking rate designed to equalize forage allowance across treatments on pastures composed of old-world bluestem (*Bothriochloa ischaemum* (L.) Keng). There were no average differences in ADG over the 3-yr period but stocking rate was doubled as N fertilization was increased from 0 kg/ha N to 170 kg/ha N. In the current study, although a fixed stocking rate was set to match anticipated forage production, the observed reduction in ADG in the 168N treatment may reflect factors beyond FM alone.

**Table 6.** Effect of dried distillers grain plus solubles (**DDGS**)^1^ supplementation and variable nitrogen (**N**)^2^ fertilization rates on animal performance of steer calves grazing wheat pastures

	Treatments^3^ (kg/ha)					
Item	100S^4^	100N	135N	168N	SEM	*P-*value^5^
Fall Grazing Season						
Grazing d	56.0	56.0	49.0	56.0	7.6	0.29
Weight gain, kg/ha	195.0	182.0	159.0	175.0	31.1	0.24
Weight, kg						
Initial	285.0	287.0	291.0	287.0	8.1	0.42
Final	364.0	360.0	356.0	358.0	7.6	0.65
ADG, kg/d^6^	1.38^c^	1.30^c^	1.26^c^	1.25^d^	0.064	0.07
Spring Grazing Season						
Grazing d	61.0^a^	48.0^b^	54.0^b^	54.0^b^	3.7	<0.001
Weight gain, kg/ha	481.0^a^	328.0^b^	354.0^b^	360.0^b^	61.5	<0.001
Weight, kg						
Initial	294.0	295.0	296.0	297.0	7.4	0.79
Final	391.0^a^	362.0^b^	367.0^b^	370.0^b^	12.3	<0.001
ADG, kg/d	1.59^a^	1.38^b^	1.32^b^	1.33^b^	0.17	<0.001
BUN^7^, mg/dL						
D0	10.5	9.69	9.69	10.0	0.366	0.34
D28	16.0^a^	10.6^b^	11.6^b^	12.8^b^	0.569	<0.001
Combined Fall and Spring Grazing Seasons						
Grazing d	117.0^a^	104.0^b^	103.0^b^	110.0^a^	9.6	0.02
Weight gain, kg/ha	676.0^a^	510.0^b^	513.0^b^	535.0^b^	63.4	<0.001

^1^Dried distillers grains plus solubles (**DDGS**) supplemented at 1.0% of steer body weight (**BW**).

^2^Nitrogen (**N**) fertilization treatments were applied yearly and split between 50% application at planting and the remaining 50% at spring green up.

^3^Treatments consisted of: 1) 100.9 kg N/ha + DDGS supplementation (100S; *n* = 6 pastures/season), 2) 100.9 kg N/ha (100N; *n* = 6 pastures/season), 3) 134.5 kg N/ha (135N; *n* = 6 pastures/season), and 4) 168.1 kg N/ha (168N; *n* = 6 pastures/season) in Yr 1 and Yr 2. In Yr 3, pastures were reduced to *n *= 5 pastures/treatment/season due to poor stand performance and insufficient available forage.

^4^100S treatment was set as control within Dunnett’s test.

^5^F-test value of analysis of variance.

^6^Average daily gain (**ADG**).

^7^Serum samples for blood urea nitrogen (**BUN**) were collected from steers (*n *= 104) in the spring of Yr 3 and analyzed via colorimetric assay.

^a-b^Within row, means with different superscripts differ at *P* ≤ 0.05 of N levels compared to Dunnett’s control.

^c-d^Within row, means with different superscripts differ at *P* ≤ 0.1 of N levels compared to Dunnett’s control.

Similar to the results of this study, a 3-yr grazing study conducted by Delevatti et al. (2019) found that the ADG of cattle grazing Marandugrass (*Brachiaria brizantha* Hochst. ex A.Rich.) was lower in bulls grazing pastures with higher applications of N fertilizer. This shows that while increasing N fertilizer may lead to greater plant growth and CP content, the relationship between N application and ADG is not always linear (Delevatti et al., 2019). The aforementioned study utilized a put-and-take grazing method, in which stocking rate is continuously adjusted to achieve optimal animal and pasture management targets. This could potentially impact pasture parameters, such as height and available mass, which could alter animal forage intake and ADG. Delevatti et al. (2019) also indicated that N application rates impacted the ratio of green leaves within swards with a lower green leaf content and higher amount of dead material observed at higher levels of N application. Research has shown that increased N application rates may diminish leaf life due to high plant growth, increasing the amount of dead material present in the pasture (Santana et al., 2017). It should be noted that the aforementioned study was conducted in tropical climates with warm season grasses as compared to the environmental conditions of this study.

Previous studies have shown that supplementation with DDGS improves ADG in beef cattle grazing forages (Morris et al., 2005; MacDonald et al., 2007; Loy et al., 2008). This was reflected in the results of this study in which the ADG of steers assigned to the 100S pastures had an increased ADG (1.59 kg/d) as compared to the other treatments during the spring grazing season. Additionally, there was a greater number of grazing d, BW gain kg /ha, and final BW (*P* ≤ 0.001) for steers assigned to 100S treatment as compared to the other treatments. For fall and spring grazing seasons combined, 100S and 168N pastures produced a greater (*P ≤* 0.05) number of combined grazing d, with 100S yielding an additional 13 grazing d compared to 100N. Furthermore, 100S steers produced a greater (*P* ≤ 0.001) combined BW gain kg/ha compared to all other treatments. When DDGS were provided to steers stocked in pastures treated with 100 kg N/ha, weight gain/ha increased by 153 kg/ha in the spring. Although there was only an additional 5 d of grazing compared to fall 100S and an additional 13 d compared to spring 100N, ADG of 100S was also 0.21 kg greater than 100N in the spring, allowing for a further increase in weight gain.

Forage mass of wheat pasture has been identified as becoming limiting for growing calves at less than 1,000 kg DM/ha (Redmon et al., 1995; Morgan et al., 2012). Forage mass may have been a limiting factor for the 100N treatment, with an initial FM of 949 kg DM/ha that decreased to a final FM of 755 kg DM/ha. This would indicate that the 100N pastures were well below the limiting threshold of 1,000 kg DM/ha for the entirety of the spring grazing season. Additionally, the increase in performance for 100S can likely be attributed to the supplementation of DDGS at 1.0% of BW. Supplementation of DDGS potentially resulted in a substitution effect, allowing for increased forage accumulation in the spring due to replacement of 1.0% of forage intake with DDGS, thus preventing limitations in FM from occurring. Moore et al. (1999) determined that energy supplementation was responsible for reductions in voluntary forage intake when forage intake was greater than 1.75% of BW, when TDN intake of supplement was greater than 0.7% of BW, or when TDN:CP was less than 7. Buttrey et al. (2012) also observed a substitution effect for stocker cattle grazing wheat pasture supplemented with either dry-rolled corn or DDGS, and their results were attributed to having a decreased TDN:CP of 3.4, which aligns with the guidelines presented by Moore et al. (1999). In the present study, all pastures from the beginning of Fall 2021 to end of Spring 2024 had an average TDN:CP of 3.32, aligning with the aforementioned studies and may explain why DDGS supplementation diminished voluntary forage intake.

### Blood Urea Nitrogen

Blood urea nitrogen was measured in spring of the final yr of the study at both grazing initiation and d 28. We were generally curious about the BUN level of 100S considering the partial substitution of high-quality forage with DDGS. In this study, BUN concentrations did not differ between treatments on d 0 with an average concentration of 9.97 mg/dL. However, BUN concentrations were greater (*P* ≤ 0.001) by a magnitude of 38% for 100S steers (16.0 mg/dL) on d 28 compared to all other treatments. This was to be expected and is similar to the results of Adams et al. (2022) who showed that BUN concentrations were greater for beef heifers supplemented with DDG as compared to heifers receiving no supplementation. Adams et al. (2022) also showed that supplemented heifers had BUN concentrations 2.7 mg/dL greater 4 hr post supplementation compared to unsupplemented heifers. In the current study, BUN of 100S steers was on average greater than BUN of unsupplemented steers ranging from 3.2 mg/dL to 5.4 mg/dL. Metabolite concentrations in the current study were greater in supplemented steers as compared to BUN concentrations reported by Adams et al. (2022) potentially due to a longer supplementation period. Blood urea nitrogen concentrations did not differ for treatments in which N was applied to pastures (*P *> 0.05), ranging from 10.6 mg/dL to 12.8 mg/dL.

In guidelines outlined by Hammond (1983) it was indicated that for steers to gain 0.5 kg/d on pasture, BUN concentrations must be between 5.0 mg/dL—25.0 mg/dL. However, maximized growth occurs with BUN values between 11.0 mg/dL—15.0 mg/dL. In relation to these guidelines, BUN concentrations for all treatments were within the range of 5.0 mg/dL—25.0 mg/dL to produce gains of 0.5 kg/d. However, cattle assigned to the 100N and 100S treatments did not fall within the 11.0 mg/dL—15.0 mg/dL range shown to produce maximized gains (Hammond, 1983). Blood urea nitrogen concentrations of 100N steers (10.6 mg/dL) fell slightly below the optimal range while 100S BUN concentrations (16.0 mg/dL) exceeded beyond the optimal range. The elevated BUN levels observed in 100S steers may be due to a synergistic effect between dietary DDGS inclusion and grazing N-fertilized wheat pastures (Holman et al., 2005). Wheat pastures, especially when fertilized with N, may provide high levels of protein to grazing cattle, potentially leading to an excess in N intake (MacKown and Northup, 2010) and an excess of urea in the bloodstream. This may be exacerbated by dietary inclusion of a high protein supplement, such as DDGS.

### Economic Results

No differences were noticed on a per ha-basis for total fall income, fall purchase cost, or total cost when cattle were supplemented with DDGS (*P *> 0.05; **Table 7**). Total spring income/ha did not differ for the 100S and 168N treatments (*P *> 0.05) but were greater (*P* ≤ 0.05) as compared to the other treatments. This indicates that in theory, supplementation of steers with DDGS grazing wheat at a lower level of N fertilization would be economically similar to steers on pastures with a higher level of N fertilization. However, numerically, the total spring income/ha of the 100S treatment was $65.67 greater than that of the 168N treatment. Additionally, the 100S treatment made an extra $82.38/ha compared to the 100N treatment. Spring income was increased for 100S as a result of an additional 13 grazing d along with an additional 0.21 kg of ADG compared to 100N. This was likely a result of additional available forage preventing DM limitations from occurring while extending the grazing season. Establishment costs were greater (*P* ≤ 0.05) for 135N ($585.00/ha) and 168N ($639.79/ha) pastures due to the added expense of the additional applied urea, while 100N and 100S establishment costs were the same as fertilizer application rates were identical ($530.22/ha). Steer management and feed costs were greater ($635.64; *P ≤* 0.001) for 100S compared to every other treatment. This was to be expected as steers on the 100S treatment were the only steers receiving DDGS supplementation. Supplementation of DDGS resulted in an increase of $514.60/ha between steers on the 100N treatment and those on 100S treatment. While total costs did not differ, total return above costs were greater (*P* *≤* 0.05) for 100N and 135N compared to 100S and 168N. Decreased returns were observed for the 100S treatment which averaged $361.00/ha less than the net return for the 100N pasture.

**Table 7.** Effect of dried distillers grains plus solubles (**DDGS**)^1^ supplementation and variable nitrogen (**N**)^2^ fertilizer rates on economic outcomes of steer calves grazing wheat pastures

	Treatments^3^ (kg/ha)					
Item	100S^4^	100N	135N	168N	SEM	*P*-value^5^
Income, $/ha						
Total fall income^6^	3,255.10	3,249.76	3,234.52	3,240.71	376.88	0.76
Total spring income^6^	7,534.38^a^	7,452.00^b^	7,443.44^b^	7,468.71^a^	1,033.22	0.03
Expenses, $/ha						
Fall purchase cost^7^	2,491.44	2,505.55	2,536.21	2,494.14	350.91	0.14
Spring purchase cost^7^	6,467.00	6,497.54	6,449.02	6,509.74	1,046.42	0.30
Establishment cost	530.22^b^	530.22^b^	585.00^a^	639.79^a^	70.42	<0.001
Calf management and feed costs	635.64^a^	121.04^b^	122.85^b^	122.18^b^	22.51	<0.001
Totals, $/ha						
Cost	9,441.48	8,997.65	9,037.26	9,109.04	964.79	0.95
Return above cost	608.06^b^	969.06^a^	906.96^a^	864.89^b^	367.95	0.009

^1^Dried distillers grains plus solubles (**DDGS**) supplemented at 1.0% of steer body weight (**BW**).

^2^Nitrogen (**N**) fertilization treatments were applied yearly and split between 50% application at planting and the remaining 50% at spring green up.

^3^Treatments consisted of: 1) 100.9 kg N/ha + DDGS supplementation (100S; *n* = 6 pastures/season), 2) 100.9 kg N/ha (100N; *n* = 6 pastures/season), 3) 134.5 kg N/ha (135N; *n* = 6 pastures/season), and 4) 168.1 kg N/ha (168N; *n* = 6 pastures/season) in Yr 1 and Yr 2. In Yr 3, pastures were reduced to *n *= 5 pastures/treatment/season due to poor stand performance and insufficient available forage.

^4^100S treatment set as control within Dunnett’s test.

^5^F-test value of analysis of variance.

^6^Income determined using a regression curve formed using data from weekly market reports for medium and large framed steers on corresponding sale dates).

^7^Purchase costs determined using a regression curve formed using data from weekly market for medium and large framed steers on corresponding purchase dates (Supplementary Table 1.).

^a-b^Within row, means with different superscripts differ at *P* ≤ 0.05 of N levels compared to Dunnett’s control.

Forage mass analysis results indicate that the substitution effect of DDGS supplementation in the spring resulted in an additional 525 kg DM/ha in comparison to 100N pastures, approximately a 35.6% increase in mean FM. Based on the additional 525 kg DM/ha of residual forage observed in supplemented pastures, it is estimated that one additional steer could be supported for every 2.81 ha. This equates to a stocking rate increase of 0.36 steers/ha, or approximately 7.2% more than the current spring rate of 4.94 steers/ha. From DDGS prices of $150.64/metric ton and upwards, supplementation is never more profitable within the pasture size used in this study and with 8 steers per pasture in the spring. However, supplementation may become economically advantageous when pasture size increases and DDGS prices are more favorable. For example, a pasture size of 18.5 ha with 6 additional steers and a DDGS price of $340.29/metric ton will break even and become more profitable than foregoing supplementation. This is supported by a study by Greenquist (2008) in which break-evens were lower for steers supplemented with DDGS and steers supplemented with DDGS grazing pastures fertilized with N. Prices of DDGS used in this study ranged from $283.12/metric ton to $372.05/metric ton. However, DDGS prices may vary as prices of DDGS from Missouri averaged $221.04/metric ton in January 2024, a decrease from an average of $280.93/metric ton in January of 2023 (USDA, 2024). While DDGS prices will continue to be subject to yearly variations in pricing and availability, prices are currently attainable, making DDGS supplementation a viable option if pasture sizes are increased.

The coefficient for converting return/ha to return/steer for this study was 0.135 based on pasture size and total steers grazed. Therefore, the average return per steer was $82.04, $130.74, $122.36, and $116.69 for 100S, 100N, 135N, and 168N, respectively. An additional economic indicator would be cost per kg BW gain, averaging $1.72, $1.28, $1.38, and $1.42/kg BW gain, respectively. As evident from the steer performance (**Table 6**) and input costs (**Table 7**), the additional cost of supplementation was too great for the relative amount of additional weight gain achieved to make DDGS more economical when stocking rate was not increased to utilize the additional spring FM with 100S. A common approach to assessing economics of supplementation would be to estimate feed efficiency or on a practical basis for cattle producers estimate feed conversion ratio (**FCR**). Supplemental FCR for fall and spring averaged 25.8 and 13.5 kg DDGS DM per kg BW gain. A limitation to supplemental feed conversion with this study is that stocking rate was fixed. Increasing stocking rate to utilize the additional FM would likely improve the FCR when expressed on an increase in BW gain/ha v. BW gain/hd.

## CONCLUSIONS

During the fall grazing season, no benefits to steer or wheat pasture performance were observed when steers were supplemented with DDGS. Supplementation of DDGS to steers grazing wheat pastures in the spring allowed for improved ADG, which may be a result of a substitution effect of DDGS on forage intake. However, it may also reflect the advancing maturity of wheat with the benefits of supplemental nutrients from DDGS becoming more pronounced as forage quality declines. This improved ADG prevented available FM in 100S pastures from decreasing to amounts that would become limiting to forage intake, similar to amounts seen in 100N pastures. Additionally, the potential substitution effect reduced voluntary forage intake, leaving an additional 525 kg DM/ha in supplemented pastures. While not evaluated in the current study due to limited pasture size, this additional forage potentially creates an opportunity for increasing stocking rate by stocking an additional steer per every 2.81 ha increase in size. However, the profitability of this in comparison to foregoing supplementation is dependent on DDGS prices, forage value, labor, and available pasture size.

## Supplementary Material

txaf116_suppl_Supplementary_Materials_1
