# Supplementary material for: Effects of variable nitrogen fertilization rates and dried distillers grains plus solubles supplementation on forage use, animal performance, and economic outcomes of steer calves grazing winter wheat pastures
Source: Transl Anim Sci. 2025 Aug 28;9:txaf116. doi: 10.1093/tas/txaf116 (PMC12448397; doi:10.1093/tas/txaf116)
Supplement: txaf116_suppl_Supplementary_Materials_1 [file txaf116_suppl_supplementary_materials_1.docx]

**Supplementary Table 1.** Sale and purchase dates of medium and large framed steers corresponding to closest steer receiving and pasture removal dates over a three-year (**yr**) period.

|  |  |  | Treatments^1^ | | | |
| --- | --- | --- | --- | --- | --- | --- |
| Study Yr | Season | Item | 100S | 100N | 135N | 168N |
| Yr 1 | Fall 2021 | Receiving dates | Oct 20 – 21 | Oct 20 – 21 | Oct 20 – 21 | Oct 20 – 21 |
|  |  | Corresponding purchase date | Nov 1 | Nov 1 | Nov 1 | Nov 1 |
|  |  | Removal dates | Dec 15 – Feb 7 | Jan 5 – Feb 7 | Dec 15 – Jan 13 | Dec 15 – Feb 7 |
|  |  | Corresponding sale date | Jan 31 | Jan 31 | Jan 31 | Jan 31 |
|  | Spring 2022 | Receiving dates | Feb 9 | Feb 9 | Feb 9 | Feb 9 |
|  |  | Corresponding purchase date | Feb 7 | Feb 7 | Feb 7 | Feb 7 |
|  |  | Removal dates | May 3 | Apr 5 – May 3 | Apr 5 – May 3 | Apr 5 – May 3 |
|  |  | Corresponding sale date | Apr 25 | Apr 25 | Apr 25 | Apr 25 |
| Yr 2 | Fall 2022 | Receiving dates | Oct 12 | Oct 12 | Oct 12 | Oct 12 |
|  |  | Corresponding purchase date | Oct 10 | Oct 10 | Oct 10 | Oct 10 |
|  |  | Removal dates | Jan 26 | Jan 10 – Jan 26 | Jan 26 | Jan 26 |
|  |  | Corresponding sale date | Jan 23 | Jan 23 | Jan 23 | Jan 23 |
|  | Spring 2023 | Receiving dates | Feb 16 | Feb 16 | Feb 16 | Feb 16 |
|  |  | Corresponding purchase date | Feb 20 | Feb 20 | Feb 20 | Feb 20 |
|  |  | Removal dates | May 10 | Apr 12 – May 10 | Apr 12 – May 10 | Apr 19 – May 3 |
|  |  | Corresponding sale date | May 1 | May 1 | May 1 | May 1 |
| Yr 3 | Fall 2023 | Receiving dates | Oct 24 | Oct 24 | Oct 24 | Oct 24 |
|  |  | Corresponding purchase date | Oct 23 | Oct 23 | Oct 23 | Oct 23 |
|  |  | Removal dates | Jan 11 – Jan 17 | Jan 11 – Jan 17 | Jan 11 – Jan 17 | Jan 17 |
|  |  | Corresponding sale date | Jan 22 | Jan 22 | Jan 22 | Jan 22 |
|  | Spring 2024 | Receiving dates | Feb 13 | Feb 13 | Feb 13 | Feb 13 |
|  |  | Corresponding purchase date | Feb 12 | Feb 12 | Feb 12 | Feb 12 |
|  |  | Removal dates | Apr 29 | Apr 15 – Apr 29 | Apr 29 | Apr 29 |
|  |  | Corresponding sale date | Apr 29 | Apr 29 | Apr 29 | Apr 29 |

^1^Treatments consisted of: 1) 100.9 kg N/ha + DDGS supplementation (100S; *n* = 6 pastures/season), 2) 100.9 kg N/ha (100N; *n* = 6 pastures/season), 3) 134.5 kg N/ha (135N; *n* = 6 pastures/season), and 4) 168.1 kg N/ha (168N; *n* = 6 pastures/season) in Yr 1 and Yr 2. In Yr 3, pastures were reduced to *n* = 5 pastures/treatment/season due to poor stand performance and insufficient available forage.
